# Compositional Consequences of Partial Dealcoholization of Red Wine by Reverse Osmosis-Evaporative Perstraction

**DOI:** 10.3390/molecules24071404

**Published:** 2019-04-10

**Authors:** Duc-Truc Pham, Vanessa J. Stockdale, David Wollan, David W. Jeffery, Kerry L. Wilkinson

**Affiliations:** 1 School of Agriculture, Food and Wine, The University of Adelaide, Waite Campus, PMB 1, Glen Osmond, SA 5064, Australia; duc-truc.pham@adelaide.edu.au (D.-T.P.); david.jeffery@adelaide.edu.au (D.W.J.); 2 The Australian Research Council Training Centre for Innovative Wine Production, PMB 1, Glen Osmond, SA 5064, Australia; 3 Treasury Wine Estates, 97 Sturt Highway, Nuriootpa, SA 5352, Australia; Vanessa.Stockdale@tweglobal.com; 4 VA Filtration, PO Box 794, Nuriootpa, SA 5355, Australia; DWollan@memstar.com.au

**Keywords:** alcohol, evaporative perstraction, dealcoholization, reduced alcohol wine, reverse osmosis

## Abstract

This study investigated compositional changes in red wines resulting from wine alcohol removal by reverse osmosis-vaporative perstraction (RO-EP) and provides insight into the physical and chemical changes in reduced alcohol wine (RAW). Trial 1 involved RO-EP treatment of three wines that were analyzed pre-treatment, post-treatment, and post-treatment with alcohol adjustment (i.e., addition of ethanol to achieve the original alcohol content). Trial 2 involved partial dealcoholization of two wines and analysis of samples collected during RO-EP treatment, i.e., wine in, wine out, retentate, permeate (pre- and post-EP treatment) and strip water. Wine color was analyzed by spectrophotometric methods, while other compositional changes were determined by WineScan, high performance liquid chromatography (HPLC) and gas chromatography–mass spectrometry (GC–MS) analyses. In general, RAWs were slightly more concentrated than pre-treatment wines, which resulted in greater color intensity and increased phenolics and organic acids. However, partial dealcoholization resulted in lower concentrations of some fermentation volatiles, particularly ethyl esters, which may reflect ester hydrolysis following ethanol removal.

## 1. Introduction

Globally, the ethanol content of wine has progressively increased over time, which has been attributed to warmer growing conditions resulting from climate change, together with improvements to viticultural management practices and winemaking techniques [1,2,3]. Concurrently, market research suggests consumer preferences are tending towards lighter wine styles (i.e., light-bodied white, rosé and sparkling wines), comprising lower levels of alcohol [4]. Winemakers are therefore employing a range of strategies to achieve wines of lower ethanol content, applied either: (i) pre-fermentation, e.g., harvesting grapes ‘early’, when sugar levels are lower [5,6,7]; (ii) during fermentation, e.g., by arresting fermentation before sugars are fully converted to ethanol [1], by fermenting with low-alcohol-producing yeast [8], or by diluting the concentration of sugar and/or alcohol through the addition of water to juice or wine [7]; or (iii) post-fermentation, e.g., by removing alcohol from wine through distillation [9,10]. For now, alcohol removal post-fermentation remains the most widely practiced approach [3]. 

Dealcoholization of wine can be achieved via means such as thermal distillation, adsorption, extraction and fractionation methods [1]. However, the two most common techniques are spinning cone column (SCC) distillation [11] and reverse osmosis combined with evaporative perstraction (RO-EP) [12]. Besides water and ethanol, these techniques do not produce waste materials, and both have been implemented at industrial scale. Furthermore, the recovery and subsequent use of ethanol generated during dealcoholization ensures these processes meet the criteria for ‘clean’ technology [9]. SCC involves heating wine, which may negatively impact wine sensory properties, and therefore quality; the capital costs associated with SCC distillation equipment are also relatively high [5,13]. 

The first patent for the application of RO for dealcoholization of beer and wine was obtained by the German brewing company Lowenbrau in 1975 [3]. Since RO membranes remove smaller molecules (e.g., water) more readily than larger molecules (e.g., ethanol), RO-treated wine can actually have higher ethanol levels than untreated wine; i.e., the addition of water is necessary to achieve dealcoholization. This has limited the application of RO, because traditionally the addition of water to wine has been prohibited (or limited) in many wine-producing countries [14]. 

Evaporative perstraction (EP) membranes have been used to remove ethanol from the permeate fraction obtained following RO treatment of wine. EP membranes have hydrophobic properties that ensure retention of a bulk liquid, whilst permitting the flux of ethanol vapor from either wine or RO permeate to a water ‘stripping’ solution. The rate at which ethanol is removed during EP depends on processing conditions such as the membrane surface area, feed flow rate, the stripping solution flow rate, and temperature, thus aroma loss can occur with prolonged treatment times and/or at elevated temperatures [15]. Nevertheless, the combined RO-EP treatment process has achieved commercial success [12] and has overcome many of the issues associated with dealcoholization methods that make use of a single membrane [16]. However, the impact on wine chemistry of the RO-EP treatment has not been well studied, particularly on an industrial scale.

This study aimed to investigate compositional changes in red wines as a consequence of partial dealcoholization (i.e., decreases of 0.5 to 5.0% alcohol by volume, (abv)), achieved via RO-EP treatment. Wines were analyzed pre-treatment, post-treatment, and post-treatment following alcohol re-adjustment (i.e., the addition of ethanol to achieve the original wine alcohol content) to determine compositional changes associated with alcohol removal. In a subsequent trial, samples were collected during RO-EP treatment to enable the composition of permeate, retentate and strip water fractions to be studied. Given the contribution of ethyl esters to the fruity aromas and flavors of red wine, this study also sought to determine to what extent dealcoholization of wine might impact ester concentrations via changes to ester-acid equilibria.

## 2. Results and Discussion

The intended outcome of RO-EP treatment is the partial dealcoholization of wine but the process may also impact other wine constituents. For example, the concentrations of non-volatile compounds such as tannins and anthocyanins, which typically have molecular weights that far exceed the molecular weight cut-off (MWCO) of RO membranes, can increase because the removal of alcohol effectively concentrates the wine by 0.5 to 5.0% [3]. In contrast, lower molecular weight compounds including wine volatiles may decrease in concentration, as compounds pass through both the reverse osmosis and perstractive membranes [3]. Chemical analysis of red wines was therefore performed before, during and after RO-EP treatment to determine compositional changes due to partial dealcoholization. 

### 2.1. Effect of RO-EP Treatment and Alcohol Re-Adjustment on Basic Wine Composition

Basic wine parameters, including alcohol, density, pH, titratable acidity (TA), volatile acidity (VA), the gelatin index (a chemical measure of astringency), and wine color measurements for Trial 1 wines are shown in Table 1. Partial dealcoholization by RO-EP achieved a significant decrease in the alcohol content of wines, being 1.6, 2.6 and 0.7% abv for wines A, B and C, respectively. Analysis confirmed the addition of ethanol to wines following RO-EP (i.e., alcohol re-adjustment) restored the alcohol content to the same levels as that of the initial wines. Differences in alcohol content did not significantly influence wine density, but significant differences in viscosity were observed amongst wine samples. In each case, RO-EP treatment of wine significantly decreased viscosity (by between 6 and 9%) which was likely driven by changes to ethanol levels, in agreement with a previous study involving partial dealcoholization of model wines [17]. The subsequent addition of alcohol increased viscosity, but not to the same levels observed prior to dealcoholization (Table 1). This might reflect the loss of some wine components through EP, such that alcohol-adjusted wines were not physically the same as pre-treatment wines.

There were no significant changes in the pH, TA, VA, or organic acid concentrations of wines as a consequence of either RO-EP treatment or the subsequent alcohol re-adjustment. However, dealcoholization significantly affected free sulfur dioxide levels. In the case of wines A and B, there was no detectable sulfur dioxide remaining after RO-EP treatment, whereas 1.9 mg/L remained in Wine C after dealcoholization. Although the initial levels of free sulfur dioxide were low, given its role in preventing oxidation and microbial spoilage of wine, this was an important finding and highlights the need for sulfur dioxide levels to be checked following RO-EP dealcoholization. As expected, there was no change in free sulfur dioxide levels following alcohol re-adjustment. 

Changes in alcohol content are thought to affect interactions between salivary proteins and wine tannins [18], influencing the perception of mouthfeel properties, including astringency. The gelatin index of wines was therefore determined as a chemical measure of astringency, with RO-EP treatment found to affect wines differently (Table 1). A significant increase was observed for wine A (from 38 to 55%, *P* < 0.001), suggesting dealcoholization would likely increase the perceived astringency, in agreement with previous studies [18,19]; whereas the 5% increase observed for wine B was not significant and there was no change for wine C. Alcohol re-adjustment significantly decreased the gelatin index values obtained for wines A and C (by 9 and 3%, respectively), but the 6% decrease observed for wine B was not statistically significant. The varied effects of (partial) dealcoholization on salivary protein interactions by wine type (i.e., variety) has previously been reported and was attributed to compositional differences besides alcohol content (i.e., wine pH, and tannin and organic acid concentrations) [18]. Similar compositional variation amongst Trial 1 wines might therefore explain why RO-EP treatment impacted the gelatin index measurements differently. 

For wines A and B, RO-EP treatment resulted in significant intensification of wine color, whereas the hue of all wines increased with dealcoholization, albeit by relatively small amounts in the case of wines B and C (Table 1). Differences in wine color following dealcoholization were further characterized by CIELab measurements, with significant differences observed for each parameter, i.e., lightness and hue intensities. However, RO-EP treatment affected the color of individual wines differently. For wines A and B, the lightness (L*) decreased by 7 and 4%, and hue intensities (a* and b*) increased by 6 and 4%, respectively; whereas for wine C, lightness stayed the same, a* decreased slightly and b* increased substantially. The subsequent addition of ethanol to dealcoholized wines did not restore the color properties of wines to those observed prior to RO-EP treatment, with alcohol re-adjustment affecting the color of individual wines in different ways. Wine C was not significantly different in color but for wines A and B, ethanol re-adjustment decreased the intensity of wine color compared to that of wine post RO-EP, presumably as a consequence of dilution. In the case of wine B, wine color following ethanol addition was even significantly lower than that observed prior to RO-EP treatment (18.8 vs. 19.6, Table 1), which was not the case for Wine A (12.2 vs. 10.8). The hue of dealcoholized wines decreased following alcohol re-adjustment compared to that of wine pre RO-EP, but to higher, lower and comparable levels for wines A, B and C, respectively. Differences in color are expected to be detectable to the human eye where ΔE* is ≥ 3.0 [20], thus, partial dealcoholization visibly affected the wine color for wines A and C (ΔE* = 5.3 and 3.0, respectively, Table 1), with a perceivable color difference remaining in wine A even after alcohol re-adjustment (ΔE* = 4.0).

As with gelatin index measurements, the variation in wine color properties observed amongst treatments likely reflected more than just the concentration or dilution of anthocyanins as a consequence of RO-EP or ethanol re-adjustment, respectively. A study involving the removal of 2% ethanol from wine by nanofiltration and reverse osmosis found the intensity of wine color increased by 6 and 11%, respectively [21], whereas the dilution of wine by the addition of ethanol decreased wine color as a result of lower co-pigmentation [22] and vice versa [23]. However, dealcoholization may also have affected the extent of co-pigmentation and/or formation of derived pigments as a consequence of oxygen uptake, the loss of sulfur dioxide and/or adsorption on the membrane surface, as suggested by Gambuti and colleagues [18]; decreased color following the addition of ethanol might be explained by the disruption of copigmentation stacks [23]. 

### 2.2. Effect of RO-EP Treatment and Alcohol Re-adjustment on Wine Fermentation Volatiles

Among the various volatile compounds formed as a result of fermentation, esters contribute many of the important fruity aromas and flavors typically found in wine [24]. Ethanol plays an important role in the acid-ethyl ester equilibrium (Equation (1)). Thus, the removal of ethanol through dealcoholization of wine could be expected to impact the concentrations of ethyl esters and their corresponding acids, due to the resulting equilibrium shift towards ester hydrolysis. Esterification and hydrolysis are reversible reactions [25], with the rate of each reaction being influenced by factors including temperature and activation energy [26], and different points of equilibrium being established for individual esters [27,28].

RCOOC_2_H_5_ + H_2_O ⇌ RCOOH + C_2_H_5_OH, (1)

To determine the impact of dealcoholization on esters, the concentrations of a range of volatile acids, esters and alcohols were measured before and after RO-EP treatment, and following alcohol re-adjustment (Table 2). RO-EP treatment of wine resulted in a decrease in the concentration of acids by as much as 50%, with higher proportions of acids with lower molecular weights, e.g., acetic, propionic, and butanoic acids, being diminished as a result of dealcoholization than acids of higher molecular weight such as hexanoic, octanoic, and decanoic acids. Indeed, decanoic acid concentrations appeared to increase with RO-EP treatment. The concentrations of branched acids similarly decreased with dealcoholization, but not significantly, and only by ≤ 10%. Theoretically, the concentration of acids should increase slightly if ester hydrolysis occurred. However, the loss of acids through EP meant that most acid concentrations decreased; for wine A, the concentrations of butanoic, hexanoic, octanoic and decanoic acids decreased by 45, 7, 1 and 25%, respectively. 

Decreases in ethyl ester concentrations were also observed, with ethyl esters of higher molecular weight, e.g., ethyl octanoate and ethyl decanoate, decreasing the most despite smaller, more volatile esters being expected to transition across the RO membrane and be removed via EP more readily. Nevertheless, the loss of ethyl esters may not only be wholly attributable to membrane filtration. The removal of ethanol might also have affected the ester equilibrium (Equation (1)), such that esters were hydrolyzed to release ethanol and their corresponding acids. 

Previous research found esters with higher molecular weights (or longer carbon chains) undergo higher rates of hydrolysis [25]. During wine aging, the concentrations of straight chain ethyl esters (i.e., ethyl hexanoate, octanoate and decanoate) were found to decrease, which was attributed to their enzymatic formation during fermentation being at levels exceeding their equilibrium concentrations [29]. A subsequent study on esterification of tartaric acid with ethanol in model wine demonstrated the importance of ethanol concentration; with an increase in both the rate and quantity of ester formation observed at higher ethanol concentrations [30]. Furthermore, esterification rates were found to decrease as molecular weight increased, i.e., ethyl decanoate > ethyl octanoate > ethyl hexanoate [26]. When two red wines with different initial ethanol concentrations (being 15.4 and 13.3% v/v) were partially dealcoholized (to remove 2, 3 and 5% *v*/*v* ethanol) using a polypropylene hollow fiber membrane contactor apparatus, ethyl ester levels decreased by between 11 and 100% [31]. Similar results were observed in a study involving dealcoholization of wine using a benchtop RO-EP system; ethyl ester concentrations decreased by 20 to 80% as ethanol concentrations decreased from 13.7% (for Shiraz wine) and 12.2% (for Chardonnay wine), to 8% (or even to 5%) [32]. Interestingly, in these studies, the concentrations of some acids increased, e.g., hexanoic and propanoic acid levels increased by 24% [31] and by 11 to 173% [32], respectively, which may reflect ester hydrolysis. 

The addition of ethanol to dealcoholized wines again had variable consequences on wine composition. The concentrations of fermentation volatiles were expected to decrease due to dilution following the addition of ethanol, and in some instances, this was observed; e.g., the concentrations of octanoic and decanoic acids decreased significantly (*P* ≤ 0.021). However, the concentrations of many volatiles did not change substantially and the levels of ethyl decanoate were found to increase significantly (*P* = 0.019). This could reflect shifts in the acid-ester equilibrium, i.e., in favor of esterification to produce ethyl decanoate. Importantly, there were very few instances in which the fermentation volatile concentrations of ethanol re-adjusted wines were comparable to those of wines prior to RO-EP treatment, which demonstrates the impact on wine aroma chemistry of the dealcoholization process. The compositional consequences of RO-EP treatment of red wines was investigated further in Trial 2, with different samples (i.e., wine, retentate, permeate and strip water) being collected during the dealcoholization process.

### 2.3. Basic Composition of Samples Collected during RO-EP Treatment of Wine

RO-EP treatment of wines D and E achieved decreases in alcohol content of 2.6 and 2.4%, respectively (Table 3). Retentate fractions generated by RO yielded a higher ethanol concentration than the initial wine in both cases, for reasons given above. That is, although both ethanol and water permeate the RO membrane, water permeates at a higher rate due to its considerably lower molecular weight. The removal of alcohol due to EP can be clearly seen by the significant decrease in ethanol content for permeate fractions before and after EP (i.e., >10% abv difference), with the ethanol subsequently being transferred to the strip water (which contained ~8–9% abv). Small but significant differences in density were observed, in particular for permeate and strip water fractions. Glycerol concentrations increased in retentate due to RO fractionation, with the lower levels observed in permeate being substantially affected by EP, despite glycerol not being detected in strip water. The levels of glycerol in wine out samples were slightly higher than in untreated wine, which may impact the perception of viscosity [33]. 

The pH of wine, retentate and permeate remained relatively consistent, despite the increased TA of retentate and decreased TA of permeate following RO as a consequence of the retention of organic acids, including succinic and lactic acids (Table 3). Notably, EP had no effect on TA which may be due to the levels of organic acids pre-EP being quite low. VA was not affected by RO-EP treatment. 

The intensity of wine color increased significantly due to dealcoholization (Table 3), but with minimal impact on wine hue, similar to that of wines A, B and C in Trial 1 (Table 1). CIELab measurements suggested the color increase was reflective of wines becoming darker, with significantly lower L* and higher a* values obtained. The color properties of retentate fractions were augmented (relative to wine samples before or after treatment) due to the concentration of anthocyanins and derived pigments during RO. This was further reflected by the complete absence of color in permeate and strip water fractions. For wine D, the ΔE* following RO-EP treatment was only 2.0, which is not expected to be evident to the naked eye, but for wine E, ΔE* was 3.7, and so the change in color would likely be perceptible. The impact of RO treatment on wine color was more obvious due to the intensification of color in retentate from both wines; with ΔE* being >10 (Table 3), this color change would also be expected to be readily observed.

### 2.4. Concentration of Fermentation Volatiles in Samples Collected during RO-EP Treatment of Wine

The concentrations of volatile acids, esters and alcohols present in wine, retentate, permeate and strip water samples are shown in Table 4. It should be noted that these concentrations do not represent absolute quantities of volatiles since the volume of wine, permeate and strip water are not the same. Therefore, mass flow values were calculated (Table 5) to better demonstrate changes in fermentation volatiles. 

It is evident from Table 4 that all of the fermentation volatiles measured were capable of permeating the RO membrane. Different proportions of each volatile were distributed in the retentate and permeate fractions but fermentation volatiles were typically present at higher concentrations in retentate compared with permeate. Following EP treatment of permeate, the concentrations of most volatiles decreased, suggesting they were being removed. This notion was supported by their detection in strip water. As a consequence, volatile concentrations in dealcoholized wines were generally lower than the levels observed in wine prior to RO-EP treatment. As outlined above, this outcome may also reflect shifts in the equilibrium between ester formation and hydrolysis due to the removal of ethanol. This was consistent with an earlier study that reported decreases in ester concentrations of up to 60% in red wines following alcohol removal using a polypropylene membrane [34]. A separate study also observed a significant loss of esters (ethyl octanoate, ethyl acetate and isoamyl acetate) following RO-EP treatment of wine to remove alcohol [35].

## 3. Materials and Methods 

### 3.1. Chemicals

Chemicals and solvents (analytical grade) were purchased from Sigma Aldrich (Castle Hill, NSW, Australia) and Merck (Darmstadt, Germany), respectively. Deuterated internal standards (d_5_-ethyl propanoate, d_5_-ethyl 2-methylpropanoate, d_9_-2-methylpropyl acetate, d_5_-ethyl butanoate, d_5_-ethyl 2-methylbutanoate, d_5_-ethyl 3-methylbutanoate, d_5_-3-methylbutyl acetate, d_5_-2-methylbutyl acetate, d_13_-1-hexanol, d_5_-ethyl hexanoate, d_13_-hexyl acetate, d_3_-2-phenylethanol, d_5_-ethyl octanoate, d_3_-2-phenylethyl acetate and d_5_-ethyl decanoate) were synthesized as previously reported [36].

### 3.2. Wine Samples

**Figure 1:**
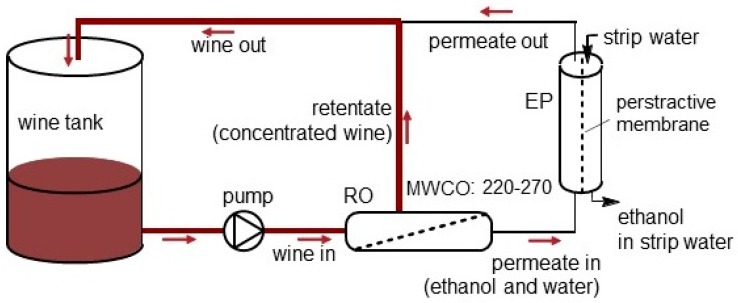
Schematic diagram of the reverse osmosis-evaporative perstraction process.

Wines were sourced from several industry partners who made use of industrial scale RO-EP units (Model Midi 10, VA Filtration, Nuriootpa, Australia) to achieve partial dealcoholization (i.e., decreases in alcohol content of between 1.0 and 2.5% abv) in accordance with the manufacturer’s operating instructions. Briefly, wines were pumped from a feed tank (industrial scale 20–30 kL) across a series of 10 spiral wound 4040 reverse osmosis membranes (nominal MWCO of 220–270 atomic mass units; filtering area 75 m^2^), under approximately 3,000 kPa of pressure, to generate retentate and permeate fractions. The permeate was degassed, heated to between 45 and 55 °C, and passed across one side of a microporous, hydrophobic hollow fiber perstractive membrane (filtering area 130 m^2^). Filtered, degassed water was passed across the other side of the membrane, as the stripping liquid. In this way, ethanol was vaporized from the permeate, diffused across the perstractive membrane and condensed in the strip water. The EP-treated permeate was subsequently cooled, recombined with the retentate and returned to the feed tank. Wine was circulated through the RO-EP unit in this way, until the desired alcohol level was achieved, as shown in Figure 1. The volume of wine in tanks was about 20–30 kL, and at this scale, industry partners found that for every 1000 L of pure ethanol removed, the wine volume decreased by 900 L, due to the mixing factor of ethanol/water. Some compounds may have been bound to the membranes, however, this would be saturated quickly at a typical flow rate of 4000 L/h. To avoid this impact, samples were collected a few hours after the commencement of processing.

Two trials involving partial dealcoholization of red wines were undertaken. Trial 1 involved RO-EP treatment of three wines: A 2014 Barossa Valley Shiraz Cabernet Sauvignon (Wine A), a 2015 McLaren Vale Cabernet Sauvignon (Wine B) and a 2015 Adelaide Hills Shiraz (Wine C). Samples were collected before and after dealcoholization and also following ethanol re-adjustment, i.e., the addition of ethanol to dealcoholized wine, to restore the initial wine alcohol content. Trial 2 involved RO-EP treatment of two wines: A 2013 Barossa Valley Shiraz (Wine D) and a 2015 McLaren Vale Shiraz (Wine E). Samples (i.e., wine in, wine out, retentate, permeate (pre- and post-EP treatment) and strip water) were collected 1.5 h after RO-EP treatment commenced. Samples were bottled in 750 mL glass bottles under screw cap closures and cellared at 15 °C prior to chemical analysis, which was performed within 2 to 3 days of RO-EP treatment. 

Since RO-EP was performed on industrial volumes, it was not practical to replicate treatments. Instead Trial 1 involved RO-EP treatment of three wines and Trial 2 involved RO-EP treatment of two wines. 

### 3.3. Basic Wine Analysis

The alcohol content, density, pH, TA (as g/L tartaric acid) and VA (as g/L acetic acid) were measured (in duplicate) by the Australian Wine Research Institute’s Commercial Services Laboratory, using a FOSS FTIR WineScan (Mulgrave, Victoria, Australia). Glycerol and organic acid concentrations were determined by high performance liquid chromatography (HPLC) using methods described previously [37]. Analyses were performed with an Agilent 1100 series HPLC (Agilent Technologies, Forest Hill, Victoria, Australia) equipped with an Aminex HPX-87H cation exchange column (Bio-Rad Laboratories, Gladesville, NSW, Australia), diode array, and refractive index detectors. The mobile phase was 2.5 mM sulfuric acid. Wine color, hue and CIELab were determined via spectral measurements made with a Cintra 4040 spectrometer (GBC Scientific Equipment, Melbourne, Vic., Australia) operating between 380 and 780 nm (at 2 nm intervals). CIELab measurements comprised: lightness (L*); the intensity of red and green hues (a*); and the intensity of yellow and blue hues (b*). The total color difference (ΔE*) was calculated according to the equation ΔE* = [(ΔL*)^2^ + (Δa*)^2^ + (Δb*)^2^]^1/2^. Viscosity was measured using an Ostwald-type viscometer (Sigma-Aldrich, 0.5 mm capillary diameter), as previously described [17], and the gelatin index of wines was measured using methodology developed by Glories, as described by Goldner and Zamora [38]. 

### 3.4. Gas Chromatography-Mass Spectrometry Analyis

The concentrations of the predominant fermentation volatiles (acids, alcohols and esters) were determined by Metabolomics Australia (the Australian Wine Research Institute, Adelaide, Australia) using gas chromatography–mass spectroscopy (GC–MS), according to stable isotope dilution analysis (SIDA) methods reported elsewhere [36]. Wine samples (1 mL, diluted with 9 mL of pH 3.7 buffer) were saturated with sodium chloride (2 g), prior to the addition of an internal standard mixture (Supplementary Table S1). Samples were extracted with a DVB/CAR/PDMS solid phase micro-extraction (SPME) fiber (Sigma Aldrich) for 10 min at 35 °C, prior to desorption (splitless mode), at an injector temperature of 200 °C, onto an Agilent 7890A gas chromatograph equipped with a Gerstel MPS2 multi-purpose sampler, and coupled to an Agilent 5975C mass selective detector. Separation was achieved with a Phenomenex wax column (60 m × 0.25 mm i.d. × 0.25 µm film thickness), with helium (Ultra High Purity) as the carrier gas (in constant flow mode). The initial oven temperature was 35 °C (held for 3 min) and then increased to 220 °C (at 5 °C/min, held for 3 min). The mass spectrometer quadrupole temperature was 150 °C, the source was set at 230 °C and the transfer line was held at 250 °C. Positive electron ionization spectra (at 70 eV) were recorded in selected ion monitoring (SIM) mode with a solvent delay of 5 min. Raw data from Agilent ChemStation software (v E.02.02.1431, Agilent Technologies, Forest Hill, Victoria, Australia) were converted into MassHunter data files and processed using MassHunter Workstation Software (Agilent Technologies) for Quantitative Analysis (v B.04.00). Fermentation volatiles were identified by comparing mass spectral data with the NIST mass spectral database and were subsequently quantified against their corresponding isotopically-labelled internal standard.

### 3.5. Statistical Analysis

Basic compositional data were analyzed by one-way analysis of variance (ANOVA) using GenStat (15th Edition, VSN International Limited, Herts, UK). Mean comparisons were performed by least significant difference (LSD) multiple comparison test at *P* < 0.05. Volatile data were analyzed via an ANOVA F-test using the lmerTest package in R statistical software [39]. For each dataset (i.e., Trials 1 and 2), mixed effect linear models were fitted individually for each volatile, with the response variable being the concentration at each treatment level. A fixed effect predictor was included for treatments, together with a random intercept for wine to account for the repeated measures on each wine. The fitting was performed using the lme4 package in R [40]. 

## 4. Conclusions

A number of options are available to winemakers to manage the final alcohol content of their wines. In this study, RO-EP was shown to effectively remove ethanol to controllable extents, but also impacted wine composition depending on both the wine in question and the amount of ethanol being removed. In general, the dealcoholization process had a concentrating effect on wines. While free SO_2_ was lost through RO-EP, some acids, and therefore TA, increased slightly but there were no significant changes to pH or VA. However, due to the contribution of ethanol to the physical properties of wine, there were significant effects of partial dealcoholization on wine color properties and the gelatin index (a chemical measure of astringency), which were attributable to changes in the concentration of anthocyanins and phenolics. Changes in ethanol concentration also affected the concentrations of ethyl esters, which may reflect both the loss of esters into strip water during EP and a shift in the chemical equilibrium responsible for ester formation and hydrolysis, following ethanol removal. From a practical consideration, winemakers need to manage the risks associated with the introduction of oxygen and loss of sulfur dioxide during RO-EP treatment, and be aware of the potential for dealcoholization to enhance wine astringency.

## Figures and Tables

**Table 1:** Density, viscosity and basic composition of Trial 1 wines before and after RO-EP treatment, and following RO-EP treatment and alcohol re-adjustment.

	Wine A					Wine B					Wine C			
	Pre-RO-EP	Post-RO-EP	Post-EtOH	*P*		Pre-RO-EP	Post-RO-EP	Post-EtOH	*P*		Pre-RO-EP	Post-RO-EP	Post-EtOH	*P*
alcohol (% abv)	14.1a	12.5b	14.1a	<0.001		17.1a	14.5b	17.1a	<0.001		14.9a	14.2b	14.9a	<0.001
free SO_2_ (mg/L)	3.8	nd	nd	–		6.4	nd	nd	–		7.7	1.9	1.9	–
density (g/mL)	0.995	0.995	0.993	–		0.993	0.996	0.993	–		0.993	0.994	0.993	–
viscosity (mPa s)	1836a	1711c	1796b	<0.001		2009a	1829c	1982b	<0.001		1858a	1751c	1836b	<0.001
glycerol (g/L)	10.2a	10.2a	10.0b	<0.001		10.9b	11.0a	10.7c	<0.001		11.9a	11.8a	11.6b	0.039
gelatin index (%)	38c	55a	46 b	<0.001		49	54	48	–		44a	44a	41b	0.040
pH	3.7	3.6	3.7	–		3.6	3.6	3.6	–		3.7	3.7	3.7	–
TA (g/L)	5.9	6.1	5.9	–		7.2	7.1	7.2	–		6.4	6.5	6.4	–
VA (g/L)	0.4	0.3	0.4	–		0.5	0.8	0.5	–		0.6	0.6	0.6	–
citric acid (g/L)	1.2	1.2	1.1	–		0.7	0.7	0.7	–		0.9	0.9	0.8	–
succinic acid (g/L)	6.2	6.2	6.1	–		8.2	8.3	8.3	–		6.5	6.5	6.4	–
lactic acid (g/L)	3.7	3.8	3.7	–		2.8	2.8	2.8	–		3.8	3.8	3.7	–
wine color (au)	10.8c	12.6a	12.2b	<0.001		19.6b	21.0a	18.8c	<0.001		17.2	17.3	17.1	–
wine hue	2.8c	4.9a	3.8b	<0.001		4.1b	4.2a	3.9c	<0.001		3.4b	3.6a	3.4b	<0.001
L*	67.6a	62.7c	64.0b	<0.001		49.7a	47.5b	49.8a	<0.001		52.9b	53.1a	52.7c	0.003
a*	34.7c	36.8a	36.1b	<0.001		50.8b	52.6a	50.9b	<0.001		49.5a	48.5b	49.6a	<0.001
b*	5.0c	5.6b	5.9a	<0.001		5.3a	5.3a	3.3b	<0.001		0.6b	3.4a	0.5b	<0.001
∆E* ^1^	–	5.3	4.0	–		–	2.7	2.0	–		–	3.0	0.3	–

Values are means of duplicate measurements (n = 2). Standard errors were ≤10%; nd = not detected. Values followed by different letters within rows (for each wine) are statistically significantly different. ^1^ Total color differences (∆E*) were calculated relative to the color of each wine prior to RO-EP treatment.

**Table 2:** Concentrations of fermentation volatiles present in Trial 1 wines before and after RO-EP treatment, and following RO-EP treatment and alcohol re-adjustment.

		Wine A				Wine B				Wine C			*P*
		Pre-RO-EP	Post-RO-EP	Post-EtOH		Pre-RO-EP	Post-RO-EP	Post-EtOH		Pre-RO-EP	Post-RO-EP	Post-EtOH	
*straight chain acids*	acetic acid ^1^	488	428	459		855	796	824		871	646	722	0.107
	propanoic acid	2009	1873	1763		1954	1846	1741		3057	2060	1838	0.190
	butanoic acid	2100	1156	1562		3399	1614	1799		2577	1514	1804	0.014
	hexanoic acid	2277	2111	2172		1757	1656	1624		1828	1778	1641	0.055
	octanoic acid	3384	3344	3075		2986	2940	2622		3016	2962	2719	<0.001
	decanoic acid	2129	2652	1870		2121	2246	1604		2066	2491	2070	0.021
*straight chain ethyl esters*	ethyl acetate ^1^	60	54	55		108	89	88		53	51	50	0.158
	ethyl propanoate	322	286	317		229	191	195		167	152	156	0.059
	ethyl butanoate	214	207	210		209	166	163		137	126	123	0.163
	ethyl hexanoate	411	385	402		375	301	300		325	294	302	0.084
	ethyl octanoate	41.8	33.8	33.7		74.0	50.5	51.8		31.5	28.0	30.5	0.145
	ethyl decanoate	114	75.7	89.0		167	93.6	109		133	96.3	116	0.019
*branched acids*	2-methylpropanoic acid	2011	1959	1818		4824	4578	4378		2300	2108	2135	0.051
	2-methylbutanoic acid	1219	1179	1206		1808	1642	1596		781	770	761	0.289
	3-methylbutanoic acid	1912	1720	1665		2337	2194	2140		1631	1625	1594	0.068
*branched ethyl esters*	ethyl 2-methylpropanoate	120	115	114		312	258	258		69.6	62.5	67.3	0.290
	ethyl 2-methylbutanoate	27.1	26.8	26.6		28.5	24.2	24.3		10.3	9.5	9.7	0.241
	ethyl 3-methylbutanoate	39.3	38.4	38.4		50.9	42.7	43.2		17.4	15.6	15.9	0.201
*acetates*	2-methylpropyl acetate	39.5	38.6	36.3		126	105	100		27.5	27.3	26.7	0.340
	2-methylbutyl acetate	356	397	338		401	334	314		179	166	162	0.335
	3-methylbutyl acetate	1140	1379	1128		1334	1111	1038		597	564	541	0.469
	hexyl acetate	24.4	31.0	13.0		12.3	9.86	9.50		8.07	6.49	6.70	0.392
	2-phenylethyl acetate	158	157	140		76.4	68.8	63.3		67.6	67.3	67.4	0.164
*alcohols*	2-methylpropanol ^1^	46	43	42		114	102	99		47	46	46	0.201
	butanol	2047	1693	1752		1232	1030	1057		1487	1340	1290	0.011
	2/3-methylbutanol ^1^	176	161	160		198	182	171		148	146	143	0.067
	1-hexanol	2695	2290	2296		2271	1977	1876		1876	1774	1744	0.027
	2-phenylethanol ^1^	78	74	73		73	70	68		55	53	52	0.003

^1^ Concentrations are µg/L, except for acetic acid, ethyl acetate, 2-methylpropanol, 2-methylbutanol and 2-phenylethyl ethanol, which are mg/L.

**Table 3:** Flow rate, density and basic composition of samples collected during RO-EP treatment of Trial 2 wines.

	Wine D								Wine E						
	Wine in	Wine out	Retentate	Permeate Pre-EP	Permeate Post-EP	Strip Water	*P*		Wine in	Wine out	Retentate	Permeate Pre-EP	Permeate Post-EP	Strip Water	*P*
flow rate (L/h)	3144	3059	2198	946	861	1138	–		3750	3664	2820	930	844	1555	–
alcohol (% abv)	15.2b	12.6d	15.9a	14.2c	3.7f	8.8e	<*0.001*		14.7b	12.3d	15.2a	13.3c	1.7f	7.6e	<0.001
density (g/mL)	0.993d	0.996c	0.997b	0.985f	0.998a	0.988e	<*0.001*		0.994d	0.997b	0.997b	0.995c	0.999a	0.990e	<0.001
glycerol (g/L)	10.0c	10.3b	12.3a	4.9e	5.4d	nd	<*0.001*		10.9c	11.3b	13.3a	4.1e	4.6d	nd	<0.001
pH	3.5c	3.5c	3.5c	3.6b	3.4d	4.0a	<*0.001*		3.6c	3.6c	3.7b	3.5d	3.4e	4.7a	<0.001
TA (g/L)	6.3c	6.6b	7.8a	2.3d	2.4d	0.1e	<*0.001*		6.4c	6.6b	7.7a	2.1d	2.1d	0.2e	<0.001
VA (g/L)	0.5	0.4	0.5	0.5	0.4	0.1	–		0.6	0.5	0.7	0.4	0.4	0.1	–
succinic acid (g/L)	5.3b	5.5b	7.2a	1.0c	1.0c	nd	<*0.001*		1.2b	1.2b	1.4a	0.6c	0.7c	nd	<0.001
lactic acid (g/L)	3.7b	3.9b	4.4a	2.5c	2.1c	nd	<*0.001*		1.8b	2.2a	2.3a	1.4c	1.3c	nd	<0.001
wine color (au)	14.8c	16.3b	20.9a	nd	nd	nd	<*0.001*		12.9c	14.2b	17.4a	nd	nd	nd	<0.001
wine hue	0.73a	0.71c	0.72b	nd	nd	nd	*0.005*		0.67	0.66	0.67	nd	nd	nd	ns
L*	57.2a	55.7b	45.9c	nd	nd	nd	<*0.001*		61.4a	58.9b	53.1c	nd	nd	nd	<0.001
a*	41.2c	42.6b	49.8a	nd	nd	nd	<*0.001*		38.2c	40.9b	44.9a	nd	nd	nd	<0.001
b*	9.6c	9.5b	13.9a	nd	nd	nd	<*0.001*		1.0c	1.3b	2.9a	nd	nd	nd	<0.001
∆E* ^a^	–	2.0	14.9	–	–	–	–		–	3.7	10.8	–	–	–	–

With the exception of flow rate, values are means of two replicate measurements (n = 2). Standard errors were ≤10%; nd = not detected. Values followed by different letters within rows (for each wine) are statistically significantly different; ns = not significant. ^1^ Total color differences (∆E*) were calculated relative to the color of each wine prior to RO-EP treatment.

**Table 4:** Concentrations of fermentation volatiles present in samples collected during RO-EP treatment of Trial 2 wines.

		Wine D							Wine E						*P*
		Wine in	Wine out	Retentate	Permeate Pre-EP	Permeate Post-EP	Strip Water		Wine in	Wine out	Retentate	Permeate Pre-EP	Permeate Post-EP	Strip Water	
*straight chain acids*	acetic acid ^1^	682	653	672	600	614	301		461	445	468	425	364	100	<0.001
	propanoic acid	2207	2264	2107	3632	3126	1423		1287	1286	1465	1010	778	401	0.417
	butanoic acid	1152	714	1289	677	<250	<250		756	736	868	589	<250	<250	0.009
	hexanoic acid	2116	2080	2488	1574	1022	728		1275	1237	1477	759	<250	<250	<0.001
	octanoic acid	2470	2283	2518	1023	529	466		1070	988	1242	587	<250	362	0.041
	decanoic acid	514	518	565	254	<100	162		493	461	542	317	207	286	0.003
*straight chain ethyl esters*	ethyl acetate ^1^	123	98	137	99	21	47		61	51	67	47	4	27	0.013
	ethyl propanoate	473	353	548	345	59	164		207	174	238	146	<25	80	0.032
	ethyl butanoate	307	239	359	183	35	92		171	145	202	85	5	47	0.004
	ethyl hexanoate	452	346	552	226	46	120		27	23	33	13	2	8	0.400
	ethyl octanoate	298	221	359	132	32	63		28	23	34	17	2	11	0.355
	ethyl decanoate	161	105	178	42	7	14		4	3	6	1	<1	<1	0.455
*branched acids*	2-methylpropanoic acid	1696	1583	1890	1084	738	506		1578	1535	1946	722	321	265	<0.001
	2-methylbutanoic acid	14586	12746	18916	5819	2474	1705		1202	1169	1506	335	177	187	0.361
	3-methylbutanoic acid	1651	1558	2141	787	447	280		1345	1311	1619	364	173	175	<0.001
*branched ethyl esters*	ethyl 2-methylpropanoate	339	307	466	121	31	52		124	102	157	41	<5	21	0.070
	ethyl 2-methylbutanoate	51	44	68	13	2	4		25	22	34	6	<1	3	0.017
	ethyl 3-methylbutanoate	82	68	111	21	3	7		37	33	48	10	<1	5	0.031
*acetates*	2-methylpropyl acetate	51	44	67	20	<5	11		45	42	58	15	<5	<5	<0.001
	2-methylbutyl acetate	191	158	244	64	11	51		106	97	140	24	<10	14	0.004
	3-methylbutyl acetate	593	474	747	202	42	181		1052	956	1345	269	23	165	0.017
	hexyl acetate	8	4	9	4	<2	5		20	18	23	10	2	6	0.142
	2-phenylethyl acetate	297	240	358	170	35	111		855	806	1040	321	39	192	0.112
*alcohols*	2-methylpropanol ^1^	68	63	85	30	7	19		66	63	81	22	2	14	<0.001
	butanol	1787	1587	1917	1458	649	989		1556	1460	1800	992	304	605	<0.001
	2/3-methylbutanol ^1^	152	143	189	59	15	38		330	339	445	158	12	80	0.037
	1-hexanol	2229	1952	2649	1262	252	761		3654	3322	4220	1900	<50	1099	0.007
	2-phenylethanol ^1^	287	280	328	171	124	43		414	409	473	238	159	67	0.001

^1^ Concentrations are µg/L, except for acetic acid, ethyl acetate, 2-methylpropanol, 2-methylbutanol and 2-phenylethyl ethanol, which are mg/L; < denotes below limit of detection.

**Table 5:** Mass flow of fermentation volatiles present in samples collected during RO-EP treatment of Trial 2 wines.

		Wine D							Wine E						*P*
		Wine in	Wine out	Retentate	Permeate Pre-EP	Permeate Post-EP	Strip Water		Wine in	Wine out	Retentate	Permeate Pre-EP	Permeate Post-EP	Strip Water	
*straight chain acids*	acetic acid ^1^	2.15	2.00	1.48	0.57	0.53	0.34		1.73	1.63	1.32	0.40	0.31	0.16	<0.001
	propanoic acid	6.94	6.93	4.63	3.44	2.69	1.62		4.82	4.71	4.13	0.94	0.66	0.62	0.001
	butanoic acid	3.62	2.18	2.83	0.64	<0.22	<0.28		2.83	2.69	2.45	0.55	<0.21	<0.39	<0.001
	hexanoic acid	6.65	6.36	5.47	1.49	0.88	0.83		4.78	4.53	4.17	0.71	<0.21	<0.39	<0.001
	octanoic acid	7.77	6.98	5.54	0.97	0.46	0.53		4.01	3.62	3.50	0.55	<0.21	0.56	0.013
	decanoic acid	1.62	1.58	1.24	0.24	<0.09	0.18		1.85	1.69	1.53	0.29	0.17	0.44	<0.001
*straight chain ethyl esters*	ethyl acetate ^1^	0.39	0.30	0.30	0.09	0.02	0.05		0.23	0.19	0.19	0.04	<0.01	0.04	0.004
	ethyl propanoate	1.49	1.08	1.20	0.33	0.05	0.19		0.78	0.64	0.67	0.14	<0.02	0.12	0.008
	ethyl butanoate	0.97	0.73	0.79	0.17	0.03	0.10		0.64	0.53	0.57	0.08	<0.01	0.07	<0.001
	ethyl hexanoate	1.42	1.06	1.21	0.21	0.04	0.14		0.10	0.09	0.09	0.01	<0.01	0.01	0.377
	ethyl octanoate	0.94	0.68	0.79	0.13	0.03	0.07		0.11	0.08	0.10	0.02	<0.01	0.02	0.319
	ethyl decanoate	0.51	0.32	0.39	0.04	0.01	0.02		0.02	0.01	0.02	<0.01	<0.01	<0.01	0.467
*branched acids*	2-methylpropanoic acid	5.33	4.84	4.15	1.03	0.64	0.58		5.92	5.63	5.49	0.67	0.27	0.41	<0.001
	2-methylbutanoic acid	45.86	38.99	41.58	5.50	2.13	1.94		4.51	4.28	4.25	0.31	0.15	0.29	0.329
	3-methylbutanoic acid	5.19	4.77	4.71	0.74	0.39	0.32		5.05	4.80	4.57	0.34	0.15	0.27	<0.001
*branched ethyl esters*	ethyl 2-methylpropanoate	1.06	0.94	1.02	0.11	0.03	0.06		0.47	0.37	0.44	0.04	<0.01	0.03	0.033
	ethyl 2-methylbutanoate	0.16	0.13	0.15	0.01	<0.01	<0.01		0.09	0.08	0.10	0.01	<0.01	0.01	0.006
	ethyl 3-methylbutanoate	0.26	0.21	0.24	0.02	<0.01	0.01		0.14	0.12	0.14	0.01	<0.01	0.01	0.009
*acetates*	2-methylpropyl acetate	0.16	0.13	0.15	0.02	<0.01	0.01		0.17	0.15	0.16	0.01	<0.01	<0.01	<0.001
	2-methylbutyl acetate	0.60	0.48	0.54	0.06	0.01	0.06		0.40	0.36	0.39	0.02	<0.01	0.02	<0.001
	3-methylbutyl acetate	1.86	1.45	1.64	0.19	0.04	0.21		3.94	3.50	3.79	0.25	0.02	0.26	0.034
	hexyl acetate	0.03	0.01	0.02	0.00	<0.01	0.01		0.08	0.06	0.06	0.01	<0.01	0.01	0.141
	2-phenylethyl acetate	0.93	0.73	0.79	0.16	0.03	0.13		3.21	2.95	2.93	0.30	0.03	0.30	0.140
*alcohols*	2-methylpropanol ^1^	0.21	0.19	0.19	0.03	0.01	0.02		0.25	0.23	0.23	0.02	<0.01	0.02	<0.001
	butanol	5.62	4.85	4.21	1.38	0.56	1.13		5.83	5.35	5.08	0.92	0.26	0.94	<0.001
	2/3-methylbutanol ^1^	0.48	0.44	0.42	0.06	0.01	0.04		1.24	1.24	1.25	0.15	0.01	0.12	0.064
	1-hexanol	7.01	5.97	5.82	1.19	0.22	0.87		13.70	12.17	11.90	1.77	<0.04	1.71	0.018
	2-phenylethanol ^1^	0.90	0.86	0.72	0.16	0.11	0.05		1.55	1.50	1.33	0.22	0.13	0.10	0.008

^1^ Concentrations are g/h, except for acetic acid, ethyl acetate, 2-methylpropanol, 2-methylbutanol and 2-phenylethyl ethanol, which are kg/h < denotes below limit of detection.

## Supplementary Materials

The following are available online, Table S1: Deuterated internal standards used for determination of fermentation volatiles by GC–MS.
