# Supplementary material for: Compositional Consequences of Partial Dealcoholization of Red Wine by Reverse Osmosis-Evaporative Perstraction
Source: Molecules. 2019 Apr 10;24(7):1404. doi: 10.3390/molecules24071404 (PMC6480466; doi:10.3390/molecules24071404)
Supplement: Supplementary file 1 [file molecules-24-01404-s001.pdf]

molecules MDPI

Table S1. Deuterated internal standards used for determination of fermentation volatiles by GC-MS.

Analyte	RT (min)	Ions[1] (*m/z*)	Internal standard
ethyl acetate	6.783	**61**, 70, 88	d_8-ethyl acetate
ethyl propanoate	8.492	75, 80, **102**	d_5-ethyl propanoate
ethyl 2-methylpropanoate	8.712	88,101, **116**	d_5-ethyl 2-methyl propanoate
2-methylpropyl acetate	10.107	**56**, 73, 86	d_9-2-methylpropyl acetate
ethyl butanoate	10.870	71, 88, **101**	d_5-ethyl butanoate
ethyl 2-methylbutanoate	11.375	85, **102**, 115	d_5-ethyl 2-methyl butanoate
ethyl 3-methylbutanoate	11.872	85, **88**, 115	d_5-ethyl 2-methyl butanoate
2-methyl-1-propanol	12.781	41, **43**, 74	d_9-2-methyl-1-propanol
2-methylbutyl acetate	13.681	57, 72, **74**	d_9-3-methylbutyl acetate
3-methylbutyl acetate	13.718	55, 87, **88**	d_9-3-methylbutyl acetate
1-butanol	14.473	41, 44, **56**	d_{10}-1-butanol
2-methyl-1-butanol	16.522	41, 56, **70**	d_9-3-methyl-1-butanol
3-methyl-1-butanol	16.522	55, 60, **70**	d_9-3-methyl-1-butanol
ethyl hexanoate	17.419	**88**, 99, 115	d_5-ethyl hexanoate
hexyl acetate	18.692	69, 73, **84**	d_{13}-hexyl acetate
hexanol	21.018	55, **56**, 69	d_{13}-hexanol
ethyl octanoate	23.441	88, **101**, 127	d_5-ethyl octanoate
acetic acid	24.058	43, **60**	d_3-acetic acid
propanoic acid	26.283	57, 73, **74**	d_5-propanoic acid
2-methylpropanoic acid	26.979	43, **73**, 88	d_7-2-methylpropanoic acid
butanoic acid	28.553	45, **60**, 73	d_7-butanoic acid
ethyl decanoate	28.811	101, 157, **200**	d_5-ethyl decanoate
3-methylbutanoic acid	29.686	**60**, 61	d_7-3-methylbutanoic acid
2-methylbutanoic acid	29.704	57, **74**	d_7-3-methylbutanoic acid
2-phenylethyl acetate	33.010	65, 91, **104**	d_3-2-phenylethyl acetate
hexanoic acid	33.435	**60**, 73, 87	d_{11}-hexanoic acid
2-phenylethanol	34.667	**91**, 92, 122	d_3-2-phenylethanol
octanoic acid	36.641	60, 85, **115**	d_{15}-octanoic acid
decanoic acid	39.304	73, **129**, 172	d_{19}-decanoic acid

[1] The bolded ion was used for quantification.

© 2019 by the authors. Submitted for possible open access publication under the terms and conditions of the Creative Commons Attribution (CC BY) license (http://creativecommons.org/licenses/by/4.0/).
